# Who Continues to a Doctoral Degree? Employment Choices and Influencing Factors Among Engineering Master’s Students

**DOI:** 10.3390/bs15091232

**Published:** 2025-09-10

**Authors:** Lina Wei, Xuejiao Wu, Min Li

**Affiliations:** 1 Institute of China Innovation and Entrepreneurship Education, Hangzhou Normal University (Key Research Center of Philosophy and Social Sciences of Zhejiang Province), Hangzhou 311121, China; 20240346@hznu.edu.cn; 2 School of Humanities and Social Sciences, Beihang University, Beijing 100191, China; xwu2022@buaa.edu.cn; 3 College of Education, Yunnan University, Kunming 650091, China

**Keywords:** engineering master’s student, learning motivation, employment, human capital accumulation

## Abstract

Career choices of master’s students, particularly regarding the pursuit of doctoral degrees, have received limited scholarly attention. This study examines the employment pathways and influencing factors of engineering master’s students. Drawing on human capital theory, career preference theory, and the two-way selection model, we analyse nationwide survey data from 21,973 engineering master’s students collected in 2021. Using a multinomial logistic regression model, we examine their post-graduation choices, including pursuing a doctorate, joining universities or scientific research institutions, working in government agencies, or entering the workforce. The findings suggest that gender, family background, and human capital have a significant impact on students’ career destinations. Male students are more likely to choose academic sectors, while those from high-income families tend to opt for non-academic sectors. Higher parental educational attainment increases the likelihood of pursuing doctoral studies, and students with more academic publications are also more likely to pursue a doctorate. These results highlight divergence in employment intentions between academic and non-academic sectors and reveal their underlying logic. The study offers insights for reforming talent cultivation models in universities, enhancing graduate employability, and informing the development of educational policy.

## 1. Introduction

Traditional postgraduate education primarily serves the talent demands within the higher education system, with the ultimate goal of ‘cultivating future researchers and university teachers’ (Griffiths, 1995). However, since the 1980s, with continuous changes in the global higher education environment and academic labour market, non-academic occupations have become increasingly central to talent cultivation in higher education (Booth, 1979). There has been a diversified trend in the destinations of postgraduate graduates. This trend first emerged in developed countries with postgraduate education, such as the United States, the United Kingdom, Germany, and France, and has become a critical challenge faced by China’s higher education in the early 21st century (Shen et al., 2015). Consequently, individuals’ motives to pursue postgraduate education have become more diverse, and this adds more uncertainty to the question ‘Who will enter the academic world?’.

Meanwhile, the discussion on ‘why and how individuals embark on an academic career path and achieve career success’ has gradually garnered widespread concern in the global academic community. Concerning this issue, most recent studies have focused on the first employment stage of doctoral students and have achieved many valuable results. However, a career choice is essentially a dynamic process, and obtaining a doctoral degree merely represents the end of one’s postgraduate education. Discussions on career choices at other stages of doctoral studies remain relatively scarce. In China’s higher education system, a master’s degree is a prerequisite for pursuing a doctoral degree; that is, the vast majority of doctoral students have already made a career choice regarding ‘whether to continue on the academic path’ before joining the doctoral stage. This provides a unique entry point for exploring postgraduate students’ career choices from a process perspective.

China’s higher education system is gradually transitioning from a mass to a popularisation model. In 2023, the number of postgraduate and graduate students recruited in China was approximately 1.148 million and 928,000, an increase of 112% and 102% compared to 2013, respectively (Ministry of Education of the People’s Republic of China, 2014, 2024). Under the general trend of overall expansion, the situations in different disciplines vary. First, among the fourteen disciplines officially classified by the Ministry of Education, the number of engineering master’s students was the largest. The number of engineering master’s students graduating each year accounts for approximately one-third of all master’s students (number of postgraduate students by discipline in 2019 and 2020). Second, the willingness of engineering master’s students to pursue a doctoral degree was lower than the overall level. A 2020 survey conducted among master’s training institutions nationwide revealed that approximately 25.3% of master’s students intended to pursue a doctoral degree (Niu & Zhou, 2021). For example, the proportion of engineering master’s students choosing to pursue a doctoral degree was approximately 14.20% (Wei et al., 2023). This might be because of the relatively large number of engineering master’s graduates and the limited enrolment quotas for doctoral programmes, or because the engineering training model has a significant practice-oriented nature (Wei et al., 2023) and it has a higher degree of integration with market demands and graduate students face more diverse employment choices. In any case, the career choice of a master’s in engineering is worth an in-depth exploration.

The career choices of engineering students are influenced by multiple factors, including individual characteristics and environmental factors (Borrego et al., 2018; M. Li et al., 2024; Mikhalkina et al., 2019; Striolo et al., 2021; Wei et al., 2023). These include gender, age, family background, motivation for enrolment, and training provided by the institution. Existing studies either consider doctoral graduates as research participants or pay more attention to students in the university or pre-university stages. Relatively few studies have specifically targeted the career choices of engineering master’s students, and national survey data are even rarer.

Based on a national survey of master’s graduates leaving school in 2021 and 21,973 questionnaires collected from engineering master’s graduates, this study explores the employment choices and influencing factors of engineering master’s students. This survey encompasses 338 postgraduate training institutions nationwide and is highly representative of the field. The study makes significant contributions to the in-depth exploration of the factors and mechanisms influencing postgraduate employment choices in academic circles. Specifically, this article focuses on the following two research questions: (1) What are the employment directions and employment intentions of engineering master’s graduates? (2) What factors influence the employment choices of engineering master’s students?

### 1.1. Literature Review and Hypothesis Development

#### 1.1.1. Influencing Factors Regarding Employment Choices for Engineering Master’s Students

1. gender Differences

Most master’s graduates are still young when they graduate, and progressing in their studies is challenging due to other socialized concerns (such as marriage and childbearing) (Niu & Zhou, 2021). Moreover, Chinese society has different expectations of the responsibilities that different genders must undertake in the family, and gender stereotypes can create structural obstacles to career development. Relevant studies examining the factors influencing the academic career development of female doctoral students in the United States found that although career alienation and the strengthening of scholarly interest during the doctoral period are important variables for predicting the choice of academic careers by female doctoral students, marriage showed a significant inhibitory effect (McClintock-Comeaux, 2006). Shao and Qu’s research found that gender stereotypes and the resulting ‘systemic discrimination’ can prevent some female master’s students from pursuing their ‘academic dreams’ (Shao & Qu, 2023). A study by Li and Sun on master’s graduates from top universities showed that the proportion of female master’s students choosing to pursue a doctoral degree was significantly lower than that of male master’s students (Y. Li & Sun, 2020). In addition, women who want to join the engineering field face resistance due to gender bias. Social stigma and the challenge of balancing work and family life deter young women from pursuing careers in engineering. Interviews conducted by Dos Santos with students majoring in electrical engineering proved this point (Dos Santos, 2022). W. Zhao and Zou (2020) also confirmed that women with a strong sense of family responsibility tend to seek career positions that require relatively less time investment and offer greater flexibility. Correspondingly, male postgraduate students are more inclined to join industries with high degrees of marketisation and higher salaries (Y. Li & Sun, 2020).

2. Family Socioeconomic Background

In Blau’s status acquisition model, the family’s socioeconomic background, specifically the parents’ educational level and occupational class, serves as an priori factor that influences the social mobility of the offspring through intergenerational transmission (Blau & Duncan, 1967). Although the government and society are committed to promoting educational equality, the current education system has not yet overcome the influence of parents’ education, occupation, and income on individuals’ educational opportunities. Research in Germany has found that in the field of mathematics, the probability of children from highly educated parents choosing an academic career is relatively high. In economics and business management, children of highly educated parents are more likely to select non-academic careers (Enders, 2002). Through a qualitative investigation, Z. Xu (2014) found that a high-quality family humanistic environment has a significant influence on the career choices of academic talents, promoting their realization of career ideals. According to Torche’s (2005) incentive hypothesis, as social inequality intensifies, people strive to compete for opportunities for upward social mobility, thereby increasing the resources they obtain. Thus, it can be inferred that children from disadvantaged families are more likely to choose academic careers, which may be due to their struggle against the social stratification of their family background. They hope to change their own and their descendants’ social classes by engaging in academic careers closely related to education.

3. Human Capital

Human capital refers to the knowledge and skills acquired through educational investment and individual efforts. Academic performance and the knowledge and skills acquired during college are typically regarded as essential indicators for evaluating an individual’s competence and future development prospects in the workplace, particularly during talent screening in the labour market. The academic award system is primarily based on Merton’s universalism principle, emphasizing professional recognition and rewards for those who are the most academically productive or who can demonstrate the most significant contributions to the discipline (Merton, 1973). Conducting more problem-based learning and actively participating in academic project practices during academic integration can significantly improve employment quality (Yu et al., 2018). Participation in academic exchange activities and social surveys is a basic measure for enhancing the professional quality of postgraduate students. This helps to integrate resources and incorporate them into students’ behavioural patterns and then constantly adjust previous goals and visions, which can promote the employment of master’s students more than publishing papers or presiding over or participating in research projects (Xie & Li, 2017). Recently, with the marketisation of higher education and the transformation of academic production models (Gibbons et al., 1994; Slaughter & Leslie, 1997), workers in academic departments have had to undertake the traditional functions of teaching and research and have to become project managers coordinating higher education and the transformation of academic production models (Gibbons et al., 1994; Slaughter & Leslie, 1997). With more postgraduate students joining the industry to undertake consulting or technological research and development work, in addition to professional qualities and scientific research capabilities, the ability to communicate and cooperate across disciplines, organizations, and countries (Hargadon & Sutton, 1997) and general and transferable abilities such as problem discovery and solving, team leadership, and project management have also become essential qualities that colleges and universities need to cultivate.

4. Learning Motivation

Admission motivation affects essential indicators of academic achievement, such as the academic cycle, depletion rate, and completion rate of engineering master’s degrees, by influencing academic attitudes. As early as 1917, Weber emphasized the importance of motivation in his speech ‘An Academic Career,’ stating that if one ‘does not devote oneself from the bottom of one’s heart to the discipline, to the discipline that enables one to achieve nobility and self-esteem through the subject one serves, then one is bound to be belittled’ (Weber, 1919/2019). Subsequent studies have also demonstrated the importance of non-intellectual factors, such as academic interest, in the career development of academic talent. Academic interest is a key factor in determining academic goals and choosing academic careers (Etmanski, 2019). Students with academic interests were more inclined to choose academic career positions. Their intention to engage in academic and scientific research was 2.6 times that of the non-interest-oriented group (Y. Zhao & Hong, 2012). Postgraduate students who aspire to a doctoral degree should possess ideals and enthusiasm for the development of the discipline (Yin & You, 2007) and must have an interest in exploring and a desire for innovation (Zha, 1999). With the expansion of scale, numerous students regard postgraduate education as a human capital investment that brings high employment rates and salaries, and their motivation for further education is no longer limited to becoming scholars (Freeman, 1986).

In summary, although some progress has been made in research on the employment status and influencing factors of master’s graduates, there are still some deficiencies, which are manifested explicitly as follows. First, concerning data samples, there is a lack of empirical data support from a large sample. Presently, research on the employment of engineering master’s students in China primarily uses samples from a particular region or university as survey objects. The sample data are limited and lacked persuasiveness in support of the research inferences. Second, concerning research subjects, there is a lack of analysis of postgraduate students in a single discipline. Currently, most research on postgraduate employment is based on surveys of all master’s graduates in different disciplines. Considering the differences in employment among master’s graduates in different disciplines, there is still a lack of in-depth research on master’s graduates in specific disciplines, especially on the employment destinations of engineering master’s graduates with apparent academic spillovers. Third, concerning the influencing factors of master’s graduates’ choice of employment, although some researchers have analysed the influencing factors of master’s graduates’ choice of direct jobs from different angles, there is still a lack of comprehensive analysis from multiple dimensions, such as individual characteristics, family background, motivation and willingness, ability accumulation, and learning experience.

#### 1.1.2. Theoretical Framework

Based on the theory of human capital, the conceptual model of career preferences (Roach & Sauermann, 2010), and the two-way selection model, this study proposes a comprehensive theoretical analysis model grounded in the conclusions of existing studies.

According to the theory of human capital, the human capital of engineering postgraduates is defined as the sum of the personal knowledge, skills, and physical strength values of college students, comprising four aspects: individual characteristics, cultural knowledge, skill levels, and comprehensive abilities. The theory of human capital explains the accumulation of human capital among engineering postgraduates and their subsequent labour productivity upon entering the labour market. It supplements the theoretical content of the characteristics of household labour supply in labour economics.

According to the career preference model, when engineering postgraduates choose whether to join an enterprise or an academic circle, the primary consideration is the degree of matching between the job attributes of the two and their interests. Engineering postgraduates who prefer scientific research and work freedom prioritize joining enterprises. According to a two-way matching theory, the employment of engineering postgraduates is a process of mutual selection between individual engineering postgraduates and employment departments. Academic communities and enterprises are the two main sectors that employ engineering postgraduates. Master’s graduates with diverse abilities, preferences, and backgrounds will thoroughly consider various factors when deciding whether to work in academia or enterprises. Similarly, the academic community and enterprises also select master’s graduates to apply for jobs based on the needs of their organizations. Eventually, the two selection behaviours reach a state of balance. The employment of engineering postgraduates is a process of mutual selection between individual engineering postgraduates and the employment departments. Therefore, the employment preferences of master’s graduates inevitably affect their employment choices and outcomes. Studies have pointed out that master’s graduates who prefer non-monetary gains (such as job independence, research interests, and job stability) when choosing a career are more likely to choose to work in academia than in enterprises (Agarwal & Ohyama, 2013).

The above two theoretical models provide a sound analytical framework for understanding whether engineering master’s students join enterprises for employment; however, they also concurrently ignore the influence of some key variables. The employment preferences of engineering master’s graduates are unachievable. For instance, some engineering master’s graduates who prefer to join the academic field may also have to consider joining enterprises owing to factors such as fewer outstanding publications and family influence. This study suggests that, when evaluating the actual employment outcomes of engineering master’s degrees, factors such as students’ individual characteristics, family backgrounds, and the accumulation of human capital during their study period should be considered. These are additional to preferences and mutual matching between individuals and employment units.

Furthermore, it is essential to recognize that these micro-level determinants operate within a broader macro-environmental context, which can directly and indirectly influence graduates’ employment decisions. The current economic climate in China, particularly the cyclical fluctuations and structural transformation of the engineering sector, shapes labour demand and alters the perceived returns to different career paths (M. Xu & Zhan, 2022). Labor market dynamics (the saturation of specific engineering subfields, regional disparities in industrial investment, and evolving recruitment standards in research institutions) can either amplify or constrain the effects of individual preferences and capabilities (Chen & Shen, 2024). From an interactionist perspective, macro-level conditions may moderate micro-level relationships. For example, a tight academic job market may compel even research-oriented students to choose industry positions. Simultaneously, robust R&D investment in the private sector may attract those with strong scientific training (Allen & Van der Velden, 2011). Therefore, the integration of macroeconomic and sectoral factors into the theoretical framework enables a more comprehensive understanding of how both structural opportunities and personal agency influence the employment trajectories of engineering master’s graduates.

Concerning the specific research design, this study examined factors such as family background, personal characteristics, motivation characteristics, ability development, and the socialization process of engineering master’s graduates as independent variables. Further, the employment direction of engineering master’s graduates was used as the dependent variable. Specifically, the employment destinations of engineering master’s graduates were classified into five types. These included pursuing a doctoral degree, attending higher education or research institutions, government agencies, other public institutions, and enterprises.

#### 1.1.3. Research Hypotheses

Based on the literature review and research framework mentioned above, the main research hypotheses of this study are as follows:

**Hypothesis** **1.** *The gender of engineering master’s students has a significant impact on their employment choices. Among them, male engineering master’s students are more likely to pursue doctoral degrees.*

**Hypothesis** **2.** *The family backgrounds of engineering master’s students have a significant impact on their employment choices. Among them, engineering master’s students with low family income levels and high paternal academic qualifications have a greater probability of pursuing doctoral studies for further education.*

**Hypothesis** **3.** *The learning motivation of engineering master’s students has a significant impact on their employment choices. Among them, engineering master’s students, who are inclined towards academic motives, have a greater probability of pursuing doctoral studies.*

**Hypothesis** **4.** *The human capital of engineering master’s students has a significant impact on their employment choices. The higher the number of papers published and the higher the professional quality of engineering master’s students, the lower the probability of pursuing a doctoral degree for further studies. The more academic activities and the stronger the general ability of doctoral graduates, the greater the likelihood of being employed by enterprises.*

**Hypothesis** **5.** *The learning experiences of engineering master’s students have a significant impact on their employment choices.*

**Figure 1:**
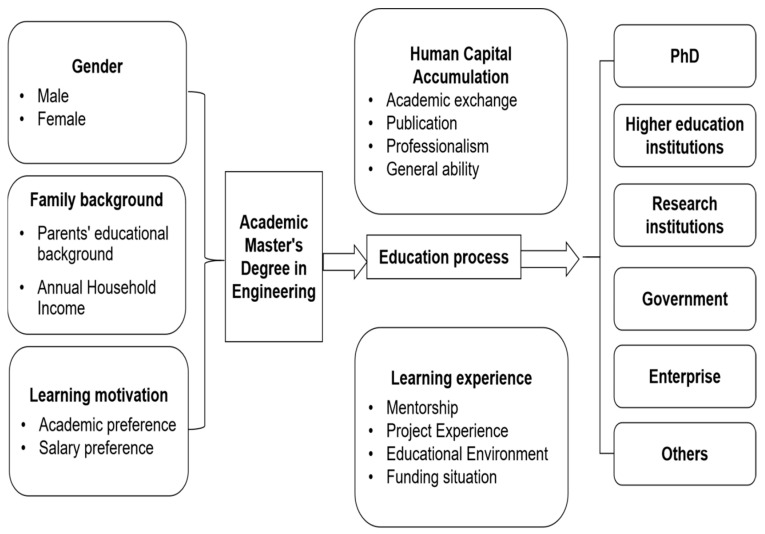
Research model diagram.

The theoretical framework of this study is illustrated in Figure 1.

## 2. Materials and Methods

### 2.1. Participants

The subjects of this survey were master’s students who were about to graduate or leave school. Concerning sampling, a stratified random sampling method was adopted to ensure the representativeness of the survey sample as much as possible. Based on the above survey sampling design, this study adopted an online survey method. The survey was conducted between May and July 2021. Survey data from 338 postgraduate training units across the country were obtained, and 70,318 questionnaires were collected, yielding a questionnaire recovery rate of 44.3%. After eliminating questionnaires with excessively short filling times and unreasonable content, 69,387 valid questionnaires were obtained, yielding an effective rate of 98.68%. Among them, 21,973 valid questionnaires were recovered from engineering master’s students. After examination, the questionnaire demonstrated good reliability and validity, and the regional and disciplinary distributions of the survey samples aligned with the overall situation of the country.

### 2.2. Variable Selection

In the framework design of this study (Table 1), the dependent variable was the employment intention of engineering master’s students. This was categorized into six groups: (1) pursuing a doctoral degree, (2) working in universities or research institutions, (3) joining government agencies, (4) joining enterprises, (5) choosing other paths, and (6) remaining undecided. The independent variables included gender; family background, such as parents’ educational attainment and family income levels; learning motivations, including preferences for academic pursuits and salary expectations; human capital, encompassing academic exchanges, paper publications, professional competence, and general abilities; and learning experience, involving mentor guidance, research project involvement, training resources, and financial support conditions. Descriptive statistics for each of the selected variables are provided in Table 1.

### 2.3. Procedure

The purpose of this investigation was to conduct a quality evaluation and problem diagnosis throughout the entire process of cultivating master’s students. Specific investigation questions were designed using questionnaires from three aspects: ‘input end’, ‘cultivation process’, and ‘output end’. Among them, the ‘input end’ mainly included the individual and family background information of engineering master’s students. The ‘training process’ primarily involved evaluating the study experience of engineering master’s students during the training period, encompassing various aspects such as supervisor guidance, course instruction, participation in research projects, academic exchanges, peer interactions, management services, and psychological support. The ‘output end’ encompassed academic gains, post-graduation destinations, and other outcomes. Based on the framework structure of the questionnaire above, the research group compiled the ‘Questionnaire for Master’s Graduates’ as a survey tool by referring to the existing questionnaires.

Data were collected between June and August 2023 using an online questionnaire distributed through university graduate schools. The survey was conducted on a voluntary and anonymous basis, with informed consent obtained from all participants. The study followed a multi-step process:

Data extraction: Engineering discipline cases meeting the inclusion criteria were selected from the full CPTQS dataset.

Data cleaning: Incomplete questionnaires, logically inconsistent responses, and cases with missing values for key variables were removed.

Variable coding: Dependent and independent variables were coded based on operational definitions; latent variables were computed using factor scores.

Dataset finalization: The cleaned and coded dataset was prepared for statistical analysis, ensuring representativeness across institution types and regions.

### 2.4. Statistical Description

The dependent variables were categorical. Therefore, the following multinomial logistic regression model was used:

(1) $$Mlogit\left(Y\right)=ln\frac{p_{i}}{p_{j}}=\alpha_{i}+\sum_{k=1}^{n}\beta_{ik}x_{k}+\mu_{i}$$

where *p_i_* represents the probability of choosing a specific employment intention; *β* is the regression coefficient, indicating the change in the ratio or the natural logarithm value caused by the change in the value of this independent variable when the values of other independent variables remain unchanged; *α* is a constant term; and *μ* is the interference term. Subscript *i* is divided into six items: ‘pursuing a doctoral degree’, ‘universities’, research institutes, or scientific research institutions’, ‘government agencies’, ‘enterprises’, ‘others’, and ‘undetermined’, and *j* = 1, with ‘pursuing a doctoral degree’ as the reference term. *X_k_* is an independent variable, and the control variable affects employment intention.

Model fit was evaluated using likelihood ratio tests, pseudo R^2^ statistics, and classification accuracy. All analyses were conducted using Stata 17 software, with statistical significance set at *p* < 0.05. Given that several variables in this study—such as financial support, academic resources, and institutional environment—were measured using Likert-scale items that reflect respondents’ subjective perceptions, these measures may incorporate both individual-level attitudes and contextual influences (e.g., institutional policies and regional economic conditions). Such a mixture of measurement levels raises the potential for clustering effects. To mitigate this concern, we included a set of institutional- and region-level control variables in all models and applied cluster-robust standard errors at the institutional level. As an additional robustness check, multilevel modelling specifications were estimated, and the results remained consistent with those reported in the primary analysis.

Furthermore, to meet the key assumptions of multinomial logistic regression, we examined the presence of multicollinearity among independent variables. Variance Inflation Factor (VIF) values for all variables were below the commonly accepted threshold of 5, and pairwise correlation coefficients did not exceed 0.70, indicating that multicollinearity was not a concern. These diagnostic procedures enhanced the reliability of the reported parameter estimates.

## 3. Results

### 3.1. Employment Flow Direction and Group Characteristics

**Figure 2:**
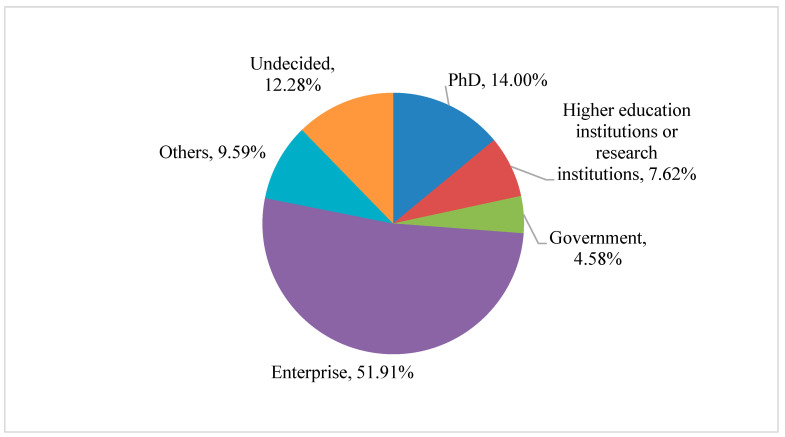
The destinations of engineering master’s students after graduation.

As presented in Figure 2, in 2021, the proportion of engineering master’s students who joined academic departments after graduation reached 21.62%. Among these, 7.62% held academic positions in universities or research institutions, and 14% of the graduates went on to pursue doctoral studies. Notably, more than 70% of engineering master’s graduates choose to join the workforce. More than half (51.91%) of the engineering master’s graduates were employed in enterprise-related positions. The remaining employment destinations included government agencies (4.58%) and other sectors (9.59%), comprising primary and secondary schools, medical and health institutions, and various public institutions. Furthermore, more than 12% of the graduates did not determine their employment destinations.

Table 2 presents the differences in human capital among engineering master’s graduates from different employment directions. Regarding academic paper publication, there were significant differences between pursuing a doctoral degree and pursuing employment. The average value of paper publications for those seeking a doctoral degree was 2.594, while for the rest it was approximately 2. Additionally, concerning professional quality, the number of graduates who visited academic departments (e.g., pursuing doctoral degrees, attending universities, research institutes, or scientific research institutions) after graduation was higher than the number of graduates from non-academic departments. Furthermore, concerning academic communication and general ability, there was no evident distribution pattern among the different graduation destinations of engineering master’s graduates. The average value of the employed group in the academic sector was significantly higher than that in the non-academic sector. Additionally, there were no significant differences in professional quality and general ability, and the average value of the employment group in the non-academic sector was slightly higher than that in the academic sector. Numerous master’s graduates with relatively high human capital spilled over to non-academic industries, reflecting the social dispersion phenomenon in the employment of master’s graduates.

### 3.2. Influencing Factors of Employment Intention

This study examines the influencing mechanisms behind the employment intentions of engineering master’s students, focusing on individual traits such as gender, family socioeconomic background, human capital, and academic interests. The results of the regression analyses are presented in Table 3.

There were significant differences in employment intentions between men and women. Male engineering master’s graduates are more inclined to pursue a doctoral degree than their female counterparts. Specifically, the probabilities of men choosing universities, research institutions, enterprises, and government agencies were 0.876, 0.792, and 0.518 times those of women pursuing a doctoral degree, respectively. Concerning family background, years of parental education had a significant impact on employment intentions. The higher the parents’ educational attainment, the more willing their children are to pursue a doctoral degree or further their studies, compared to those who attend universities, research institutions, or enterprises. With the increasing annual income of families, engineering master’s graduates are more inclined to choose employment than to further their studies. This might be because the expected monthly salary of engineering master’s graduates from families with better economic conditions is relatively high. Therefore, they tended to choose non-academic departments with relatively better wages.

Learning motivation has a profoundly positive impact on the decision to pursue an academic career. The higher the academic preference, the more inclined the engineering master’s graduate was to choose further studies. The higher the salary preference, the more inclined students are to be employed rather than pursue a doctoral degree.

Concerning human capital, the more engineering master’s students publish papers, the more likely they are to pursue further studies. This may stem from the emphasis placed on students’ publication of articles for further education. Professional quality has a significant impact on employment intention. Compared to employees, engineering master’s students are more willing to continue exerting their professional abilities and devote themselves to academic pursuits. Master’s graduates with strong general abilities tend to choose overseas postdoctoral research as their career development direction. Scholarly communication and general ability also affect the employment choices of engineering master’s students; however, both have the reverse effect on students’ tendency to pursue a doctoral degree. For instance, for every additional unit of academic exchange, the probability of an engineering master’s graduate choosing employment is approximately 1.2 times that of pursuing a doctoral degree.

Concerning learning experience, the frequency of supervisors guiding students has no significant impact on the employment choices of engineering master’s students. Project experience significantly increases the probability of master’s students pursuing engineering doctoral degrees. Among them, this impact is more evident than employment in non-academic organizations (government agencies and enterprises). Furthermore, both training conditions and financial aid significantly increased the possibility of students choosing direct employment.

## 4. Discussion

This study empirically analysed the factors influencing the employment intentions of engineering master’s students, further clarifying the impact of gender, family background, and human capital, among others. The relevant findings reveal the characteristics and differences among master’s graduates with different career intentions, which are conducive to reflecting on training objectives and related policies for engineering master’s students.

The multinomial logistic regression results (Table 3) further illuminate the differentiated determinants of career intentions among engineering master’s graduates. First, destination patterns are highly skewed toward non-academic sectors, with 51.91% choosing enterprises and only 14% intending to pursue doctoral study (Figure 2). Consistent with Niu and Zhou (2021), the odds of choosing enterprise over doctoral study are significantly higher for males (β = 0.234, *p* < 0.1), while their odds of selecting government over doctoral study are notably lower (β = −0.657, *p* < 0.01). These opposite-signed coefficients reinforce the dual nature of gender effects: men are overrepresented in industry-oriented transitions but underrepresented in public-sector pathways.

Second, family background effects exhibit a nuanced pattern. Parents’ educational background positively predicts choosing higher education or research institutions over doctoral study (β = 0.067, *p* < 0.1), yet it exerts no significant effect on choices for enterprise or government. The positive sign suggests that higher cultural capital encourages academic-adjacent careers, aligning with Bourdieu’s (1986) theory of reproduction and findings by Qiu and Xiao (2011) and Yang (2006). By contrast, annual household income coefficients are slight, positive, and non-significant across models (β range: 0.016–0.027), which diverges from prior studies (e.g., Niu & Zhou, 2021), suggesting strong economic capital effects—possibly due to China’s doctoral funding and subsidized housing systems reducing economic barriers for low-income students.

Third, human capital disparities strongly shape academic versus non-academic trajectories. Academic exchange experience significantly increases the likelihood of both enterprise (β = 0.253, *p* < 0.01) and government (β = 0.264, *p* < 0.01) employment compared to doctoral study, suggesting its role in enhancing employability in applied contexts (Xie & Li, 2017; Yu et al., 2018). In contrast, research publications have consistently negative coefficients for non-academic outcomes (e.g., enterprise vs. PhD: β = −0.567, *p* < 0.01; government vs. PhD: β = −0.367, *p* < 0.1), underscoring their function as a gateway to doctoral entry and academic careers (Shao & Qu, 2023; Cai, 2020; S. Zhao et al., 2018).

Finally, coefficient magnitudes indicate that project involvement (β = 0.172–0.171, non-significant) and educational environment ratings (β = 0.334–0.264, *p* < 0.01) have weaker or more context-dependent effects. Notably, the significant negative coefficient for academic motivation in higher education or research vs. PhD (β = −1.055, *p* < 0.01) suggests that those with a strong academic orientation are less inclined toward research positions without pursuing a doctorate, reinforcing the role of doctoral study as a threshold for sustained academic engagement.

Altogether, these findings highlight the interplay between socio-demographic, cultural, and capability-based factors in shaping the career decisions of engineering master’s graduates. The sign and magnitude of coefficients—particularly the contrast between positive exchange experience effects for industry/government and negative publication effects—underscore the need for differentiated training strategies that simultaneously sustain doctoral pipelines and strengthen non-academic career readiness.

### 4.1. Theoretical Contributions

#### 4.1.1. Innovation in Research Perspective

Focusing on the key decision-making points in pre-doctoral education. In the field of academic research, the past literature has focused relatively intensively on the initial employment choices of doctoral graduates (Chen & Shen, 2024), while paying insufficient attention to the key decisions made during the pre-doctoral education stage. This study ingeniously shifts the perspective forward, anchoring on the key career decision-making node that master’s students face: whether or not to pursue a doctoral degree. Under the framework of China’s higher education system, the master’s stage, as the core preparatory period for doctoral education (Shao & Qu, 2023), plays a decisive role in subsequent academic career development. A vast majority of postgraduate students must choose their pursuit intentions and paths at this stage (W. Xu & Cen, 2021). This shift in research fills the gap in academic research during this critical transitional period. Furthermore, it is beneficial to thoroughly analyse the internal mechanisms that influence the career decisions of master’s students, thereby clarifying the key factors that shape the talent cultivation chain from master’s to doctoral degrees. It also provides a new perspective and research direction for optimizing the talent cultivation path in higher education and enhancing the career development guidance mechanism.

#### 4.1.2. Breakthroughs in Research Object and Data

Empirical analysis of an engineering master’s group based on a large-scale sample. This study demonstrates remarkable innovation and validity regarding the selection of research subjects and data support. Contrary to the previous generalized selection of research participants (Hao et al., 2023; Xie & Li, 2017), this study targeted a group of engineering master’s students with diverse and complex career development paths. Owing to the characteristics of engineering majors, this group faces both the possibility of delving deeply into the academic field and pursuing a doctoral degree to explore the technological frontier, including the multiple choices of joining enterprises, government agencies, and other institutions to apply theoretical knowledge in practice. The factors influencing their career decisions are more diverse, and their research value is more prominent. The study conducted a national empirical analysis based on large-scale survey data covering 338 training units across the country and contained 21,973 valid observations. Such large-scale and nationwide representative sample data not only effectively compensate for the lack of empirical evidence regarding the career choices of this specific group in previous studies, but also provide a solid data foundation for accurately analysing the career decision-making model of engineering master’s students and exploring the deep influencing factors, greatly enhancing the universality and reliability of the research conclusions. This provides strong data support for the formulation of relevant policies and practical guidance.

#### 4.1.3. Advances in Theoretical Framework

Building a comprehensive multidimensional analysis system. Concerning the construction of a theoretical framework and the expansion of analytical dimensions, this study achieved a significant breakthrough. Abandoning traditional investigation methods that focus on single or limited dimensions in research, this study innovatively integrates theories of human capital (Schultz, 1971), occupational preference, and two-way selection. This helps construct a comprehensive and systematic multidimensional comprehensive analysis framework. This framework systematically explores the comprehensive influence of various factors on the career choices of engineering master’s students (covering multiple directions, such as pursuing a doctoral degree and joining universities or research institutions, enterprises, or government agencies) from various perspectives, including individual characteristics, family background, learning motivation, human capital accumulation, and learning experience. Organically integrating elements such as individual intrinsic traits, family environmental factors, academic motivation, and ability accumulation overcomes the limitations of previous studies in the dimension of variable examination and provides a more comprehensive and detailed explanatory mechanism for a deeper understanding of the career decision-making behaviour of engineering master’s students, which is conducive to revealing the complex influencing mechanisms behind career choices. It promotes the deepening and improvement of theories related to career development in higher education, providing new theoretical paradigms and analytical ideas for subsequent research.

Furthermore, by linking macro-level structural trends to micro-level behavioural mechanisms, the proposed framework offers a more contextually grounded explanation of why similar individual profiles may yield divergent career outcomes under different economic and industrial conditions. This deepens theoretical interpretations of career decision-making in higher education and enhances the practical relevance of research findings for policymakers and institutional leaders aiming to align graduate training with evolving labour market demands (F. Li et al., 2011).

It promotes the deepening and improvement of theories related to career development in higher education, providing new theoretical paradigms and analytical ideas for subsequent research.

### 4.2. Practical Implications

#### 4.2.1. Policy Recommendations

Drawing on the multinomial logistic regression results from 21,973 engineering master’s students (2021), several policy actions can better align talent pipelines with diversified employment destinations while sustaining doctoral supply quality. First, equity-oriented doctoral pipeline policies are needed. Given that parental education is positively associated with doctoral continuation and that students from higher-income families are more likely to choose non-academic sectors, ministries and provincial authorities should expand need-based scholarships and living stipends targeted at academically strong, low-income, and first-generation students. Doing so could reduce financial deterrents to doctoral entry and counterbalance the background effects evidenced in the data. Second, early, research-intensive preparation should be incentivised. The demonstrated link between research output (e.g., publications) and the decision to pursue a doctorate suggests creating national or regional schemes that fund research assistantships, conference travel, and manuscript-development workshops within master’s programs. These instruments strengthen human capital that is empirically predictive of doctoral continuation. Third, gender-responsive measures are warranted given men’s higher propensity to enter academic sectors. Targeted mentoring, bridge fellowships, and family-friendly funding rules (e.g., flexible timelines and parental leave extensions on scholarships) could improve women’s access to academic research careers without compromising standards. Fourth, consistent with the two-way selection perspective, balanced support across sectors should be maintained. Doctoral fellowships and postgraduate research positions could be designed in partnership with enterprises and government research units (e.g., industrial PhD fellowships and applied research residencies). This would enable students with strong research interests but heterogeneous career goals to progress without being hindered by sectoral disparities.

#### 4.2.2. Implications for Higher Education Institutions

Universities and graduate schools can translate the findings into program-level practices. First, adopt a segmented, data-informed approach to career advising. Map advising services to the four observed destinations (doctoral study, universities or research institutes, government agencies, and enterprises). Implement aspiration ‘check-ins’ in the first year to identify doctoral intent early and tailor mentoring accordingly. Second, provide targeted support for first-generation and low-income students—including doctoral-literacy workshops (admissions mechanics, funding structures, and supervisor matching), application coaching, and fee-waiver or grant navigation—to mitigate the background advantages associated with parental education and income. Third, strengthen research training and publication pipelines within master’s programs through, for example, structured RA assignments, small seed-grants for publishable capstones, writing bootcamps, and faculty-led publication studios. These mechanisms operationalise the paper’s result that research output correlates with doctoral continuation. Fourth, implement gender-sensitive mentoring and transparent progression criteria in research groups to ensure equal access to high-impact projects and authorship opportunities, thereby addressing the observed gender gap in academic trajectories. Fifth, expand cross-sector research pathways (e.g., co-supervised theses with industry and government labs and short doctoral internships) so that the two-way selection between students and institutions is based on informed fit rather than information asymmetry or resource constraints.

### 4.3. Limitations and Future Research

First, this study was limited by its cross-sectional data, which were based on the 2021 National Master’s Graduate Survey. While it captures career choice patterns at a specific time point, it does not reflect the dynamic, evolving nature of students’ decision-making throughout the entire postgraduate journey. Factors such as research output, industry trends, and shifting personal goals may change across the stages of a study (Guo et al., 2023; Z. Xu, 2018; Yao, 2025), and static data cannot fully reveal these temporal shifts or their underlying mechanisms.

Second, the variable measurements were not exhaustive. Although multiple dimensions were considered, indicators for human capital and learning experience, such as paper count and project participation, failed to capture deeper qualities, such as research depth, innovation, team collaboration, and the application of general competencies (e.g., communication and problem solving). Future studies should refine the measurement tools and develop more comprehensive index systems to better reflect these constructs.

Third, the finding that household economic capital has a negative correlation with doctoral pursuit intentions contradicts the existing literature. Although plausible explanations exist, such as financial aid systems and stronger upward mobility motives among economically disadvantaged students, these hypotheses lack empirical validation. Future research could adopt qualitative methods (e.g., interviews) or longitudinal tracking to explore how family background, cultural atmosphere, and social support interact to shape doctoral aspirations.

## Figures and Tables

**Table 1:** Variable definitions and statistical descriptions.

Variable	Sample Size	Mean Value	Standard Deviation	Explanation
**Dependent variable**				
Employment intention	21,335	/	/	Including six categories: PhD, higher education institutions or research institutions, government, enterprise, others, undecided
**Independent variable**				
Gender	21,728	0.640	0.480	Female value = 0, male value = 1
**Family background**				
Parents’ educational background	21,840	4.680	1.859	Convert the educational background of the father and mother into years of education
Annual household income	21,973	4.190	1.570	Annual total income of the family (including bonus subsidies, etc.)
**Learning motivation**				
Academic preference	21,973	3.542	0.770	2 observation questions, 1–5 points (1 = strongly disagree, 5 = strongly agree)
Salary preference	21,973	4.300	0.718	The purpose of pursuing a master’s degree is to focus on high-paying jobs
**Human capital accumulation**				
Academic exchange	21,866	3.847	0.668	5 observation questions, 1–5 points (1 = strongly disagree, 5 = strongly agree)
Publication	21,973	2.074	0.865	Foreign academic journal articles + domestic academic journal articles × 0.4
Professionalism	21,973	3.991	0.745	5 observation questions, 1–5 points (1 = strongly disagree, 5 = strongly agree)
General ability	21,973	3.936	0.745	10 observation questions, 1–5 points (1 = strongly disagree, 5 = strongly agree)
**Learning experience**				
Mentorship	21,973	7.333	1.271	Mentor (offline + 0.5 × online) guidance frequency
Project experience	21,973	3.090	1.323	Number of projects participated in
Educational environment	21,973	3.927	0.757	School software and hardware conditions
Funding situation	21,973	3.878	0.761	Scholarships and other school subsidies

**Table 2:** Comparison of human capital indicators for engineering master’s graduates from different occupations.

	Human Capital Accumulation			
	Academic Exchange (St.d)	Publication (St.d)	Professionalism (St.d)	General Ability (St.d)
PhD	3.837 (0.700)	2.594 (1.258)	4.088 (0.715)	4.009 (0.718)
Higher education institutions or research institutions	3.922 (0.655)	2.030 (0.804)	4.072 (0.729)	4.039 (0.721)
Government	3.900 (0.652)	2.070 (0.816)	4.022 (0.746)	4.044 (0.738)
Enterprise	3.862 (0.658)	1.974 (0.717)	4.016 (0.724)	3.953 (0.729)
Others	3.862 (0.646)	1.996 (0.726)	3.971 (0.738)	3.936 (0.740)

**Table 3:** Analysis of a multivariate logistic regression model for the employment of engineering master’s graduates.

Variable		Higher Education Institutions or Research Institutions vs. PhD (Probability Ratio)	Enterprise vs. PhD (Probability Ratio)	Government vs. PhD (Probability Ratio)
**Constant**		1.114 ***	4.984 ***	0.119
		0.300	0.212	0.368
**Gender**	Gender	−0.133 * (0.876)	−0.234 *** (0.792)	−0.657 *** (0.518)
		0.069	0.049	0.079
**Family background**	Parents’ educational background	−0.068 *** (0.934)	−0.167 *** (0.847)	−0.036 (0.964)
		0.019	0.014	0.023
	Annual household income	0.067 *** (1.069)	0.067 *** (1.070)	0.145 *** (1.156)
		0.023	0.016	0.027
**Learning motivation**	Academic preference	−1.055 *** (0.348)	−1.405 *** (0.245)	−1.536 *** (0.215)
		0.052	0.039	0.059
	Salary preference	0.233 *** (1.262)	0.289 *** (1.335)	0.669 *** (1.952)
		0.054	0.039	0.066
**Human capital accumulation**	Academic exchange	0.173 ** (1.189)	0.253 *** (1.287)	0.261 *** (1.298)
		0.068	0.048	0.082
	Publication	−0.493 *** (0.611)	−0.567 *** (0.567)	−0.367 *** (0.693)
		0.037	0.023	0.044
	Professionalism	−0.261 *** (0.770)	−0.058 (0.944)	−0.512 *** (0.599)
		0.083	0.058	0.097
	General ability	0.185 ** (1.203)	0.000 (1.000)	0.551 *** (1.735)
		0.087	0.060	0.104
**Learning experience**	Mentorship	0.034 (1.203)	0.016 (1.016)	−0.022 (0.978)
		0.027	0.019	0.032
	Project experience	−0.006 (1.034)	−0.089 *** (0.915)	−0.117 *** (0.889)
		0.023	0.017	0.029
	Educational environment	0.314 *** (1.369)	0.219 *** (1.245)	0.162 ** (1.176)
		0.066	0.045	0.076
	Funding situation	0.171 *** (1.187)	0.163 *** (1.176)	0.119 (1.126)
		0.063	0.043	0.075

Note. *, **, and *** indicate the significance at 0.1, 0.05, and 0.01 levels, respectively. The figures in parentheses represent the probability ratios, and the row below them is the standard error.

## Data Availability

The data will be made available upon request.
